# The metallurgical characteristics of non-precious alloys using Nd:YAG laser welding

**DOI:** 10.1186/s40824-015-0047-x

**Published:** 2015-12-03

**Authors:** Jun-Hee Lee, Seok-Kyu Choi, Min-Ho Hong

**Affiliations:** Department of Advanced Materials Engineering, Dong-A University, Busan, Korea; Department of Bio-medical Research Institute, Kyungpook National University Hospital, Daegu, Korea

**Keywords:** Non-precious alloys, Laser weld, Hardness, Microstructure

## Abstract

**Background:**

This study aimed to determine the effect of hardness change according to penetration depth in the laser fusing zone and observed the correlation of the microstructure as an Nd:YAG laser was irradiated to Ni-Cr alloy for dental use by setting the spot diameter size with respect to defocusing distances. In all groups, the hardness depth profiles in the laser fusing zone and heat-affecteded zone (HAZ) had larger values than those of the base metal. In addition, the hardness values in places beyond the fusing zone and the HAZ were measured as being quantitatively lower.

**Methods:**

The alloys used in this study were Verabond 2 V, Noritake Super, and Bellabond Plus, which are commercially used non-precious dental alloys. The specimens were cut to have a plate shape with a size of 0.5 × 3.0 × 2.5 mm. This was followed by setting the Nd:YAG laser output, pulse duration, and frequency to 60 W, 10 ms, and 5 Hz, respectively. The laser was then irradiated as the spot diameter condition varied between 0.5 mm and 1.4 mm in accordance with defocusing distance from 0.0 mm to 2.0 mm. After the laser irradiation, a cross-section of the fusing zone in the specimens was observed in terms of laser melted depth, hardness depth profile, and the microstructure of each alloy.

**Results:**

The observation result of the diffusion of the constituent elements and microstructure using field emission scanning electron microscopy, energy dispersive spectroscopy (EDS), and electron probe micro-analyzer showed that the fusing zone revealed a much finer dendritic form than the base metal due to the self-quenching effect after laser melting, while no change in constituent elements was found although some evaporation of the main elements was observed.

**Conclusions:**

These results suggest that each Mo and Si combined inter-metallic compounds were formed on the interdendritic area. Through this study, the laser fusing zone had better hardenability due to the inter-metallic compound and grain refinement effect.

## Background

The importance of joining the connecting parts of casts for prosthesis manufacturing in the dental field has increased significantly. Welding is required when metals need to be joined for a variety of reasons, such as re-attachment of the severed prosthesis or fractures in order to fix the deformation or errors after casting during the manufacturing of the metal structures of the fixed prosthesis [1].

The soldering methods generally used in existing dental fields inevitably introduce gas into the soldering connecting part, thereby leaving bubbles and degrading the strength of the connecting parts; using gas also results in the formation of a wide heat-affected zone (HAZ), which can change the particle size of the joint metals and decrease their tensile strength. It can also cause increased corrosion due to the direct contact between the deposited metals and the other different types of soldering materials, resulting in the reduction in strength of the joint parts. These shortcomings in the existing soldering methods can be overcome using Nd:YAG laser welding [2, 3].

Since laser welding concentrates energy on a small area, it creates a small HAZ. It is also performed under an Argon atmosphere so that it has the advantage that oxidation is minimized in the area surrounding the diameter where the laser is irradiated [4]. As such, laser welding has been proven to be a more effective technology than existing soldering methods [5, 6].

When laser welding is performed, some of the laser beams are reflected from the surface of the metal, while the rest of the beams are absorbed into the metal. Only those laser beams that are absorbed are used for material processing. The amount of laser beam absorbed into the surface of the metal can be different depending upon the wavelength of the laser beam surface roughness and the thermal conductivity of the metal. The absorbed part is changed into heat energy from the metal surface, thereby producing an important heat source for laser welding [7].

Once the laser heat source that is converged by the laser beam is irradiated to the surface in the pre-determined axis direction, only a local layer on the surface is heated by the thermal conductivity that occurs inside, and the heat on the surface is rapidly cooled down as soon as the laser beam is removed. Such a cooling process is known as self-quenching [8].

It has been known that the fusing zone and the surrounding HAZ experience changes in both their microstructure and hardness at the penetrated fusing zone due to the rapid cooling [9]. While this type of change in the metallurgical characteristics due to laser irradiation has been reported, this study aims to discuss the metallurgical characteristics that are changed by Nd:YAG laser welding used in the casting of a dental alloy by setting a laser spot diameter size with respect to defocusing distances, which can be selected generally in the laser welding.

## Methods

### Materials and specimen preparation

VeraBond 2 V (Aalbadent Inc., USA), Noritake Super(Noritake Dental Supply Co., Limited, Japan), and Bellabond plus (Bego, Germany), which are commercialized Ni-Cr alloys for dental use, were employed for specimen manufacture (Tables 1 and 2).

**Table 1:** Chemical compostion of dental alloys (wt. %)

Dental alloys	Ni	Cr	Mo	Ga	Nb
Verabond 2 V	71.8	12.8	9	-	4
Noritake super	59.6	23.5	9.22	0.46	-
Bellabond plus	65.2	22.5	9.5	-

**Table 2:** Material properties dental alloys

Alloy	Verabond 2 V	Noritake super	Bellabond plus
Density (g/cm^3^)	8.2	8.0	8.2
Vickers hardness (Hv)	373	335	200
Yield strength (MPa)	754	507	420
Elongation (%)	18 %	15 %	12 %
Tensile strength (MPa)	1021	699	560
Melting range (°C)	1290–1335	1180–1245	1325–1370
CTE(×10–6/°C) (25 °C ∼ 500 °C)	13.7 × 10–6 · K-1	13.4 × 10–6 · K-1	13.9 × 1010–6 · K-1

Once the specimens were cut to have an acryl plate shape with a size of 0.5 × 3.0 × 2.5 mm^3^, followed by investment and burnout according to manufacturer’s instruction, material casting was performed using a highfrequency casting machine (SuperCast 3; SEIT, Italy) within the cast temperature range for each alloy. After the casting was complete, the investment material was removed. Then, steam cleaning was performed on the specimens by spraying 110 μm Al_2_O_3_ from a distance of 10 mm with a pressure of 3 bar for 10 s.

### Laser beam irradiation and measurement of penetration depth

The laser was irradiated to the center of the specimens under the same condition, with an output of 60 W, pulse duration of 10 ms, and frequency of 5 Hz set in the Nd:YAG.

Laser (Galileo Manifredi Laser welder, Manfredi, Italy), and the condition of spot diameter was changed between 0.5 mm and 1.4 mm in accordance with defocusing distance from 0.0 mm to 2.0 mm (Table 3). The spot diameter of fusion zone were examined using optical microscope (SZX7; Olympus, Japan).

**Table 3:** Condition of laser irradiation test (*n* = 3)

Spot diameter (mm)	Power (W)	Pulse duration (ms)	Frequency (Hz)
0.5	60	10	5
0.8			
1.0			
1.2			
1.4			

In order to measure the laser melted depth, the fusing zone was cut into half using a metal cutting machine (Sapcom; Hyunyang Co., Ltd, Korea) to see the crosssection.

Once the cross-section of the specimen is placed facing the base, molding was performed using a mounting press (MOT-FTA-2; F-tech Co., Korea), and mirror polishing was conducted on #400, #800, #1200, and #1500 SiC paper using a polisher (POL-FTA-2; F-tech Co., Korea). The surface shape of the specimen irradiated after mirror polishing was observed using an optical microscope.

In addition, this study analyzed the experimental data of each material by using the statistical program called SPSS for Windows 18.0 (SPSS INC, Chicago, IL, USA) in order to identify the impact of change of laser diameter on penetration depth. The findings of penetration depth for the laser diameter of each group were recorded as mean and standard deviation. Moreover, this study examined each coefficient through simple regression analysis.

### Hardness measurement

To observe the hardenability of the alloys for dental use irradiated by a laser, changes in hardness were examined. Hardness measurement was conducted for 10 s with a 200 gf load using a micro-vickers hardness tester (HM112; Mitutoyo, Japan). By setting the 35 μm inner side from the central surface of the laser fusing zone to the origin, every 100 μm of depth up to 1300 μm was measured.

### Metallographic examination

To conduct metallographic observation after laser irradiation and alloy mirror polishing, the surface of the metal base was etched using a reagent of 7.5 mL HF, 2.5 mL HNO_3_, and 200 mL methanol, followed by observation of the specimen cross-sections using a scanning electron microscope (field emission scanning electron microscopy (FE-SEM); LEO SUPRA 55; Carl Zeiss, Germany). The acceleration voltage and current conditions of FE-SEM were 10 keV and 10 nA, respectively, and the main observation scale was within a range of 40–250. During the SEM analysis, the surface of the specimen was gold coated and analyzed with energy-dispersive spectroscopy (EDS) qualitatively (in-lens detector + secondary ion detector). In addition, element diffusion of the specimen was observed through a backscattered electron (BSE) detector (topography and composition image) by irradiating electron beams accelerated with 15–30 keV by an electron probe microanalyzer (EPMA) (JXA-8100; JEOL, Japan) to determine the zone change by means of the diffusion of the specimen elements due to laser welding.

## Results

Nd:YAG laser beam was irradiated to Ni-Cr alloys for dental use by changing the spot diameter between 0.5 and 1.4 mm, thereby measuring the laser melted depth as a result of experiment.

### Penetration depth by change in laser spot diameter

**Fig. 1:**
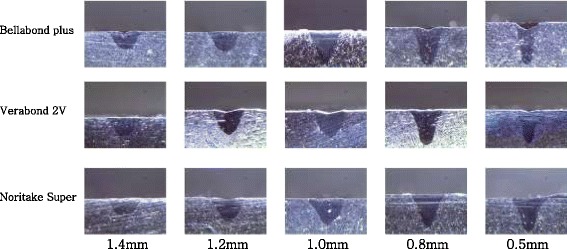
Images of cross sections at spot diameter 0.5–1.4 mm on laser power 60 W

The results showed that the Bellabond plus alloy had 0.53–1.27 mm of laser melted depth, the VeraBond 2 V alloy had 0.48–1.25 mm of laser penetration depth, and the Noritake Super alloy had 0.31–1.03 mm of laser penetration depth (Table 4 and Fig. 1). The smaller the laser diameter was, the deeper the laser melted depth was in all the specimens under the same laser output. The penetration depth of the alloys in order was Bellabond plus, VeraBond 2 V, and Noritake Super alloys.

**Table 4:** laser melted depth of laser irradiation on cast plate specimens (mm)

Spot diameter	Penetration depth [Mean ± SD]		
	Bellabond plus	Verabond 2 V	Noridake super
0.5 mm	1.27 ± 0.08	1.25 ± 0.08	1.03 ± 0.06
0.8 mm	1.17 ± 0.11	1.16 ± 0.09	0.9 ± 0.11
1 mm	1.09 ± 0.19	0.78 ± 0.15	0.58 ± 0.04
1.2 mm	0.73 ± 0.04	0.64 ± 0.05	0.46 ± 0.04
1.4 mm	0.53 ± 0.21	0.48 ± 0.07	0.31 ± 0.10

### Hardness depth profile analysis

**Fig. 2:**
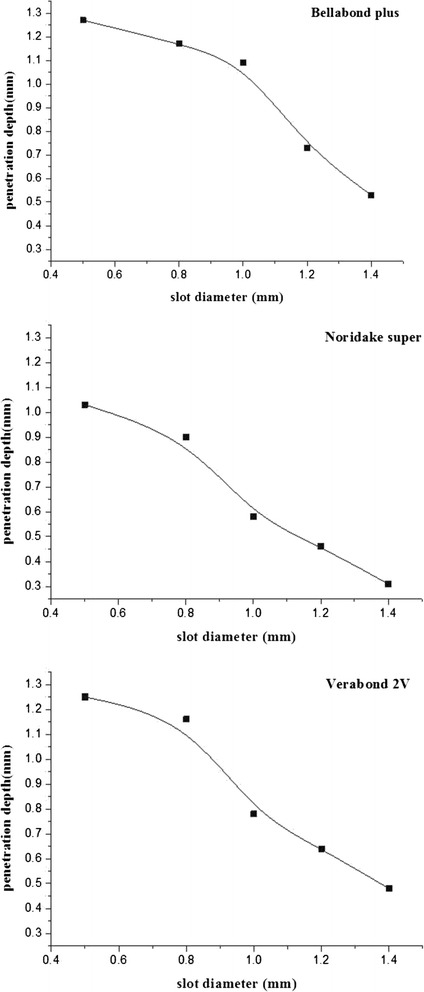
Plot of laser melted depth profiles according to spot diameters

As shown in (Table 5) and (Fig. 2), the difference in laser melted depth in accordance with the laser spot diameter for each sample group was calculated and displayed in the form of relational expression. This study examined each regression coefficient through simple regression analysis. As a result thereof, significance probability (P) was less than 0.05. Thus, a regression model was formed. Moreover, R square value was close to 1. Thus, it was verified that laser diameter would affect penetration depth. The following equations were generated through least squares method based on the aforementioned experimental data.

**Table 5:** Comparison of laser melted depth profiles according to spot diameters

Specimens	Equation (y = aχ + b)	R square	*P*
Bellabond plus	−0.851 × + 1.792	0.895	0.015
Verabond 2 V	−0.924 × + 1.767	0.944	0.006
Noridake Super	−0.847 × + 1.486	0.964	0.003

$$ \mathrm{Bellabond}\ \mathrm{plus}\ \mathrm{y}=-0.851\upchi \kern0.5em +\kern0.5em 1,792 $$

$$ \mathrm{Verabond}\ 2\mathrm{V}\ \mathrm{y}=-0.924\upchi \kern0.5em +\kern0.5em 1,767 $$

$$ \mathrm{Noridake}\ \mathrm{Super}\ \mathrm{y}=-0.847\upchi + 1,486 $$

Where, y represents laser melted depth, whereas χ represents spot diameter of laser.

No significant difference between the samples. Also, laser melted depth becomes shallower when laser diameter becomes larger.

**Fig. 3:**
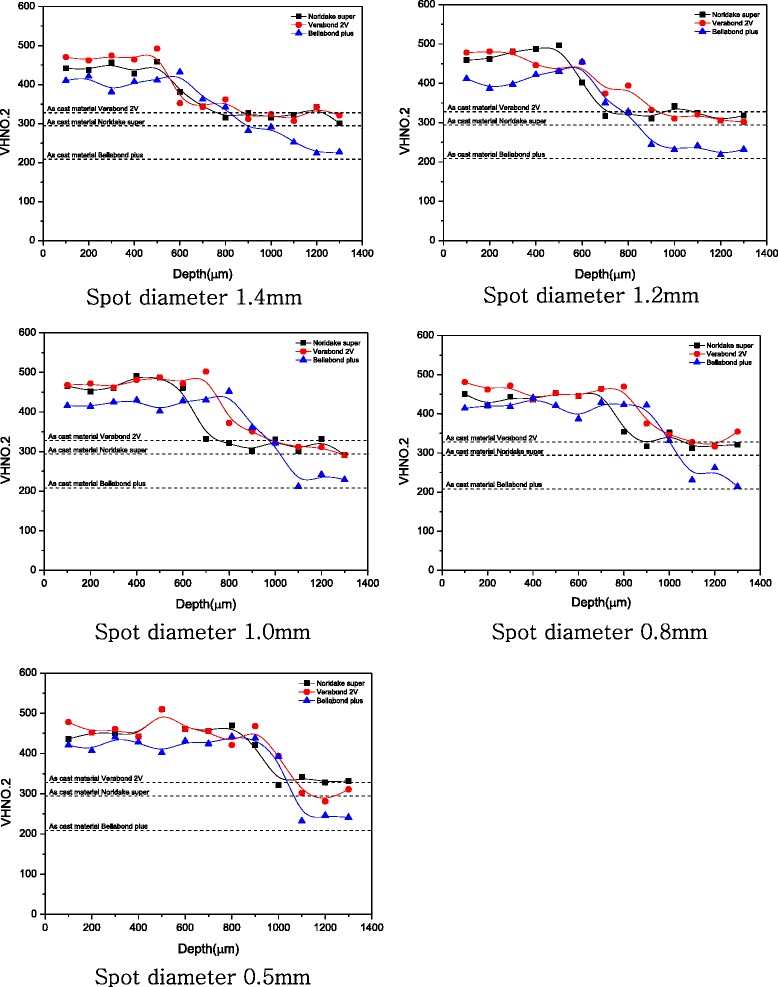
Comparison of hardness depth profiles at 35 um from cast surface to 1300 um in depth

The hardness values for each change in laser diameter are shown in (Fig. 3). In all the groups, the hardness value of laser melting area and heat-affected area was 50 % higher than the hardness value of base metal. Moreover, the hardness value was measured to be quantitatively low at a point beyond the melting area and heat-affected area.

### Microstructures

**Fig. 4:**
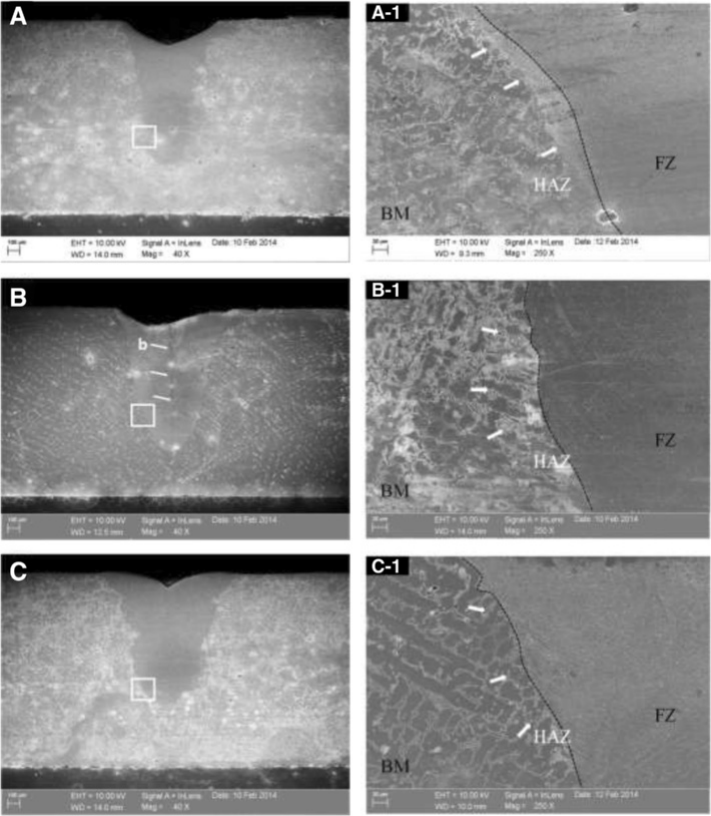
FE-SEM images of the microstructure at 0.8 mm of the Laser spot diameter. **a** Noritake super alloy; **b** Bellabond plus alloy; and **c** Verabond 2 V alloy specimens. Boundaries area of the laser irradiation: (**(a)-1**), (**(b)-1**) and (**(c)-1**); higher magnification view of the box in magnification, *b* hot tear area marking

(Figure 4) shows the cross-sectional images of the microstructure for the three different types of alloys when the laser was irradiated at 0.8 mm of the laser spot diameter. A keyhole type of penetration and melting shape was observed in the cross-sectional images of the microstructure of the (A), (B), and (C) alloys. Defects such as irregularity of structure, spatter, and hot tear area due to laser penetration, as shown in the (B) alloy, were found. At the boundary areas of the laser irradiation of each alloy for (A)-1, (B)-1, and (C)-1, three distinctive parts, the fusing zone, the HAZ, and base metal, were observed, and a fusing line that created a boundary between the fusing zone and the HAZ was identified. Although the morphology of the grains of three different types of alloys was revealed differently, the fusing zones in all the three different types of alloys did not corrode easily by the etchant in contrast with the base metal, while the fusing zone showed a much finer dendritic form than the base metal due to the selfquenching effect after welding. The HAZ showed a structure with both fine grain HAZ (FG HAZ) and inter-critical HAZ (IC HAZ). Furthermore, the base metal was not affected by the laser heat source directly as an adjacent metal area, displaying a sub-critical HAZ (SC HAZ) structure.

## Discussion

This study reviewed hardness change according to penetration depth in the laser fusing zone and also observed the microstructure while irradiating an Nd:YAG laser to an Ni-Cr alloy for dental use with a variety of spot diameter sizes.

The results revealed that as the laser sport diameter size became smaller, the penetration depth increased and a keyhole shape was displayed distinctively in all three different types of alloys. The reason for this result is that the focused spot diameter increased as the laser melted diameter decreased, and the temperature of the fusing metal exceeded the boiling point, resulting in fusion as the laser density supply was raised [10].

The change in hardenability according to penetration depth showed that the dominant factor that determines the hardness of the alloys was not decided by a hardenability function in general but by the penetration depth of heat due to laser beam irradiation [11].

That is, high hardness can be achieved because the microstructure of the base metal was modified by the laser irradiation [12]. The hardness values displayed at the fusing zones of all specimens were larger than those provided by the manufacturer of the alloys as well as those at the casting state. On the other hand, a low hardness value was found around the base metal, except in the fusing zone and HAZ. This result was found because a major structural change in grain refinement occurred in the proximity of where fusion was generated due to a high temperature, thereby creating the FG HAZ and IC HAZ of the grain in the HAZ. The lower hardness values found in the base metal beyond the fusing zone and the HAZ were found because that area was not directly affected by the laser heat source, so the area had the characteristics of the SC HAZ.

**Fig. 5:**
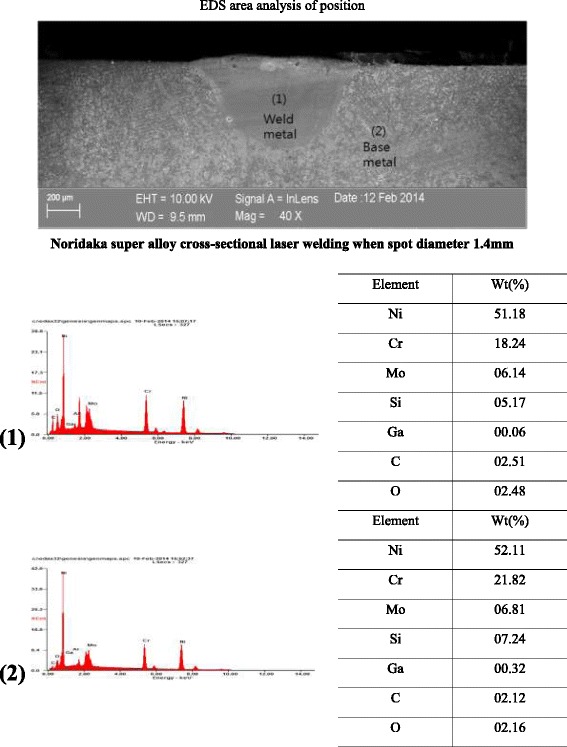
Results of EDS analysis in matrix area of fusing zone and base metal

**Fig. 6:**
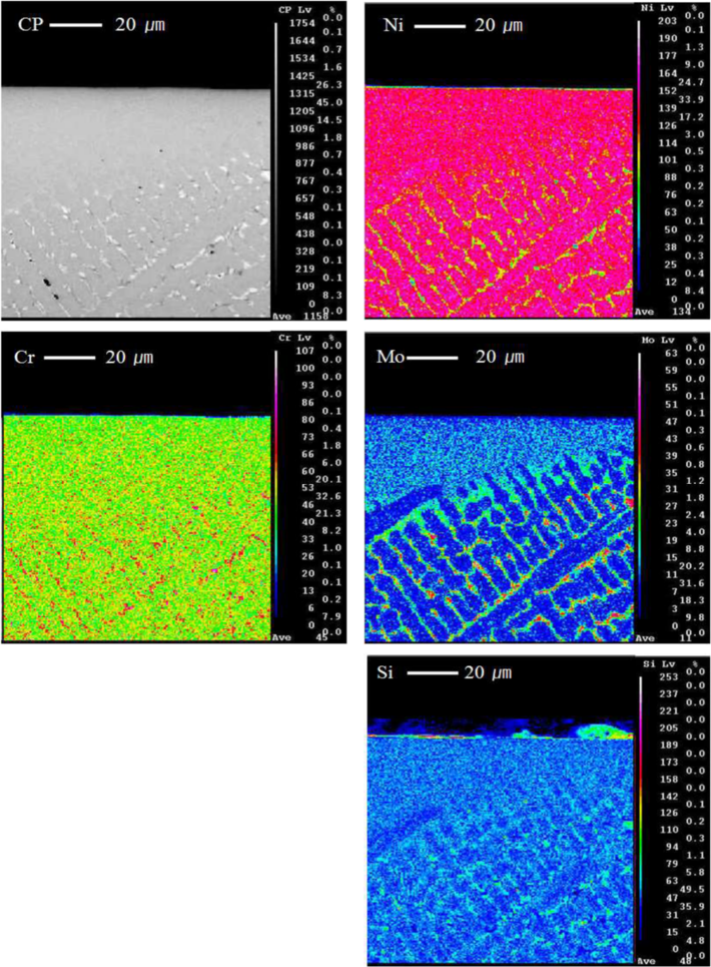
Results of EPMA analysis in matrix area of fusing zone and base metal

Regarding the improved hardenability in the welding area, a previous study by Bertrand et al. [13] estimated that a change in the composition around the welding area was minimal, but a small amount of chromium was evaporated and Mo and Si were distributed over the interdendritic area in the form of intermetallic compound. (Figure 5) shows the elemental composition analysis result of Noritake Super alloy, which represented the elements of the fusing zone and base metal qualitatively. Although a change in the composition between two zones in all types of alloys due to laser welding was not found, some main elements were evaporated a little, while the other two types of alloys had the same pattern. The result of EPMA X-ray mapping analyses, which were to determine the change in the zone due to the element diffusion, showed that Mo- and Si-combined intermetallic compound was formed over the interdendritic area, which was estimated to be a factor that improved hardenability in the welding area. Most elements in the three types of alloys had no segregation, while elemental homogenization was observed regardless of the microstructure and welding area (Fig. 6).

The grain refinement in the welding area was found because the grain refinement effect due to a cooling speed difference by the thermal stress occurred in the surface and the inside at the time of heating and cooling rather than a cooling speed difference due to the structure-sensitive property. Such grain refinement effect caused an increase in the dislocation density, thereby showing high hardness in contrast with the base metal. The reason for the formation of the fusing zone and the HAZ was that the laser-irradiated area in the alloy was heated rapidly followed by rapid selfquenching due to the surface heat conducted to the inside after a laser beam was passed through, creating nonequilibrium solidification rather than equilibrium solidification.

This study aimed to observe a correlation between hardness change and changes in metallurgical characteristics during laser welding. When a laser is irradiated to a unit area with the same energy, the penetration depth is dependent on the characteristics of alloy such as specific values of thermal conductivity and diffusion rate of the alloy, reflectivity during laser welding, and an optical absorption rate. The effect of these factors cannot be determined by the results of the experiment in this study only.

Therefore, additional experiments are necessary to understand the effect of the thermal conductivity and diffusion rate of the alloy due to laser welding in the future in parallel with identification of corrosion in the fusing zone.

## Conclusions

We observed the correlation of metal structure and reviewed hardness change by different melted depth of laser fusing zone. And we did this by investigating spot diameter size (under various conditions) of Nd:YAG laser on dental Ni-Cr alloys used for dental clinics. The important conclusions of this study are as follows:

1. The laser melted depth of all alloys increased when the diameter size of laser spot got smaller. The hardness value of melting area and heat-affected area was 50 % higher than the hardness value of base metal.
2. Although there was no change in ingredient of both fusing zone and base metal, there was intermetallic compound combined with Mo and Si elements in the interdendritic area.
3. As a result of this study, it was possible to understand that hardenability gets better by intermetallic compound and grain refinement effect on the laser fusing zone.
